# Plants' Contributions to People Shift With Glacier Extinction

**DOI:** 10.1002/pei3.70041

**Published:** 2025-04-18

**Authors:** N. Khelidj, S. Balestra, M. S. Caccianiga, B. E. L. Cerabolini, D. Tampucci, G. Losapio

**Affiliations:** ^1^ Institute of Earth Surface Dynamics, Faculty of Geosciences and Environment University of Lausanne Lausanne Switzerland; ^2^ Department of Biosciences University of Milan Milan Italy; ^3^ Department of Biotechnologies and Life Sciences University of Insubria Varese Italy

**Keywords:** Alpine flora, biodiversity conservation, deglaciation, ecosystem management, ecosystem services, Nature's Contributions to People, plant biodiversity

## Abstract

Alpine environments are among the most vulnerable ecosystems to climate change, with glacier retreat rapidly altering plant communities, biodiversity, and ecosystem functions. However, the socio‐economic consequences of these biodiversity changes remain largely unexplored. Understanding Nature's Contributions to People (NCP) provides a valuable framework for assessing biodiversity's role in human well‐being. While NCP has typically been studied at the landscape level, we focus on species‐specific contributions of plants to highlight the importance of glacial biodiversity for people. Our novel concept of Plants' Contributions to People (PCP) provides insights into the ecological, social, and economic significance of plant biodiversity and offers a practical approach for guiding conservation efforts and policy decisions. We surveyed 99 plant species in four glacier environments in the Italian Alps; one glacier (Trobio) underwent a complete extinction in 2023 while another glacier (Amola) has a widespread surface debris cover and is proximate to extinction. We then grouped plant species into early, intermediate, and late depending on their successional stages, and then linked plants to 13 different PCP based on extensive literature research. By comparing present and projected future scenarios, we assessed the absolute and relative changes in PCP under glacier extinction. Our results show that changes in PCP are primarily driven by declining plant species richness. Most affected PCP are associated with air quality, soil health, and nutrient regulation, which decrease by sevenfold on average across plant species. Whereas natural hazards regulation showed no significant variation, association with pest and disease increases especially for late species. While future plant communities may provide PCP that are qualitatively similar to present‐day communities, the volume of species‐specific contributions would decrease due to biodiversity loss associated with glacier extinction. Our results provide the first evidence of PCP shift toward erosion following a decrease in plant species richness. This case study demonstrates that PCP is a valuable tool for assessing the ecological and socio‐economic consequences of biodiversity change, helping raise awareness of the biodiversity crisis and inform conservation actions aimed at sustaining ecosystem functions in a rapidly changing world.

## 1 Introduction

In the past decades, climate and land use changes have dramatically degraded Earth environments (Mace et al. 2018; Soeder 2021). Notably, environmental degradation affects first and foremost the ecosystems on which human societies depend for a good quality of life (Adla et al. 2022). Despite many attempts to flatten the curve of biodiversity loss, rates of species extinction are currently higher than at any other point in human history, and on average 100–1000 times higher than background rates (Mace et al. 2018; Pimm et al. 2014). Recent estimates indicate that about 30% of the species have been threatened or driven to extinction since 1500 (Isbell et al. 2023). The same estimation is obtained when focusing solely on plant species (Isbell et al. 2023). In the future, we know that many more species will be threatened by extinction (Bellard et al. 2012; Trisos et al. 2020). With the global loss of biodiversity, Nature's Contributions to People (NCP) are expected to be jeopardized too (Ramel et al. 2020; Reid et al. 2006; IPBES 2019). As our society relies on nature for ecosystem services (MEA 2005) such as food provisioning, the societal consequences of species loss and ecosystem degradation have become increasingly raising concerns among scientists and policy makers (Moreira et al. 2024; Neugarten et al. 2024). Yet, the benefits of plant species to people and therein the risks to societies due to biodiversity loss remain poorly quantified on a species‐specific basis.

One way of quantifying plant–people interactions is through the frameworks of Ecosystem Services or Nature's Contributions to People (de Groot et al. 2002; Díaz et al. 2018; MEA 2005; Stenseke and Larigauderie 2018; IPBES 2019). The concept of ecosystem services (ES) includes the ecological functions that are directly beneficial to humans. It has been advocated in a science–policy context as a science‐based framework for informing policy making and to raise awareness on the societal consequences of biodiversity loss. The concept of ES was redefined in 2018 as Nature's Contribution to People (NCP) by the Intergovernmental Science‐Policy Platform on Biodiversity and Ecosystem Services (IPBES) (Díaz et al. 2018). IPBES classification considers three main types of NCP: regulating, material, and non‐material contributions (Díaz et al. 2018; Brauman et al. 2020). Regulating contributions include pollination, seed dispersal, soil formation, and soil protection. Material contributions could be the provisioning of food, forage, or timber. Non‐material contributions include artistic inspiration or scientific purposes (Díaz et al. 2018). These contributions can be positive or negative to human quality of life (Díaz et al. 2018). However, the consequences of current climate change on NCP remain poorly understood as we lack estimates of how climate change–driven biodiversity loss would affect NCP.

Plants are the foundation of terrestrial ecological systems and represent the most important part of biomass worldwide (Bar‐On et al. 2018). In doing so, plants provide a range of positive NCP, from carbon sequestration to medicines (Dal Cero et al. 2014; Godswill 2019; Weiskopf et al. 2024). For instance, plants in urban systems support NCP as trees decrease heat, air pollution, and noise pollution, or meadows encourage social activities while crops can provide food sources (Sia et al. 2023). Plants are also at the basis of our pharmaceutical medicine as most of the drugs are derived from plants, in addition to being used in traditional medicine (Howes et al. 2020; Dal Cero et al. 2014). Unfortunately, there are also examples of negative contributions, for instance, invasive species or vectors of pests (Gallardo et al. 2024; Paini et al. 2016). Yet, the delivery of NCP by plants is often approximated at a very coarse scale by considering habitats or land‐cover types (Díaz et al. 2018; Chaplin‐Kramer et al. 2019; Martín‐López et al. 2019; Külling et al. 2024). This way, evaluation and value assessments of NCP often overlook species‐specific contributions (DelSesto 2020; Roches et al. 2021; Molina‐Venegas et al. 2021; Rey et al. 2023), ignoring community variation and heterogeneity. On the contrary, valuing and quantifying plant‐based NCP on a species‐specific basis would be more informative for local biodiversity maintenance to deliver targeted actions for species conservation and for integrating botanical knowledge with local ecological knowledge (Díaz et al. 2018; Martín‐López et al. 2019). Given that not all plant species provide the same contributions but species contribute in different ways to NCP (Rey et al. 2023), and given that current NCP assessments are based at the habitat or landscape levels while ignoring species‐specific contributions, it follows that there is a lack of knowledge on the role of diverse sets and broad ranges of plant species in NCP. This knowledge gap hinders our ability to assess how plant diversity and biodiversity change influence NCP, ultimately limiting prompt conservation and restoration actions in the face of climate change.

Here, we introduce the concept of Plants' Contributions to People (PCP) defined as the species‐specific positive contributions, or benefits, and occasionally negative contributions, losses, or detriments, that plants have in socio‐ecological systems. It echoes the original idea of NCP (Díaz et al. 2018) and goes further by making explicit the relationship between plants and contributions on a species‐specific basis. It particularly considers plant diversity and the nuances of botanical knowledge.

In this context, one of the striking consequences of climate change is the retreat of glaciers worldwide (Zemp et al. 2019). Alpine landscapes are rapidly changing as glacier retreat affects downstream ecosystems and functional diversity (Bosson et al. 2023; Cauvy‐Fraunié and Dangles 2019; Meire et al. 2023; Wilhelm et al. 2013; Khelidj et al. 2024). Following glacier retreat, new areas are open for colonization and ecological succession on glacier forelands (i.e., land adjacent to retreating glaciers within the Little Ice Age moraines). Colonization processes and patterns on glacier forelands by plants and other living organisms have been widely studied in the past decades (Cantera et al. 2024; Erschbamer and Caccianiga 2016; Ficetola et al. 2021; Gobbi et al. 2010; Losapio et al. 2021; Rosero et al. 2021). Pioneer communities are established in forelands in 20–50 years following glacier retreat, leading to a rapid increase in biodiversity (Bayle et al. 2023; Cantera et al. 2024; Ficetola et al. 2021, 2024). With the proceeding of ecological succession, after about 50–100 years, species turnover becomes the most prevalent process as specialists and pioneer species are mainly replaced by generalist, competitive species (Cauvy‐Fraunié and Dangles 2019; Losapio et al. 2021; Cantera et al. 2024) while cold microhabitats (microrefugias) provided by glaciers disappear, increasing the risk of local extirpation (Gentili et al. 2020).

Even though biodiversity generally increases within 170 years of succession (but see Stibal et al. 2020; Tu et al. 2024; Anthelme et al. 2022; Erschbamer 2007), there is a substantial loss of glacier specialist and pioneer species (Anthelme et al. 2022; Losapio et al. 2021; Erschbamer and Caccianiga 2016). Provided that glaciers support unique biodiversity that is currently at risk, and given that previous studies identified current and projected states of ecosystem services provided by glacial areas as concerning (Cook et al. 2021; Palomo 2017; Zimmer et al. 2023; Bosson et al. 2023; IPCC 2019), it is reasonable to expect that NCP and PCP would be negatively affected by glacier extinction. However, there is no knowledge on the degree to which NCP/PCP are affected by biodiversity loss consequent to glacier retreat and extinction. To the best of our knowledge, no study has previously provided a species‐specific analysis on the NCP/PCP delivered by plant diversity and their fate following glacier extinction. Quantifying how NCP/PCP change with glacier retreat and extinction is key to provide science‐based evidence for informing conservation, management, and policy in light of the extreme rates at which glaciers are retreating.

In this case study, we assessed the response of PCP following glacier retreat across pioneer, intermediate, and late plant species in four Alpine glacier forelands. We hypothesize that the extinction of glaciers will erode PCP as biodiversity shifts to more generalist species while local plant diversity is decreasing. We ask the following questions: (1) What are the PCP provided by glacier foreland ecosystems? (2) How does glacier extinction affect PCP across species? Our case study not only shows the importance of NCP in glacier foreland ecosystems, but it also illustrates how species‐specific NCP assessment can be implemented and scaled to diverse plant communities and environments.

## 2 Methods

### 2.1 Study Sites and Plant Communities

**FIGURE 1:**
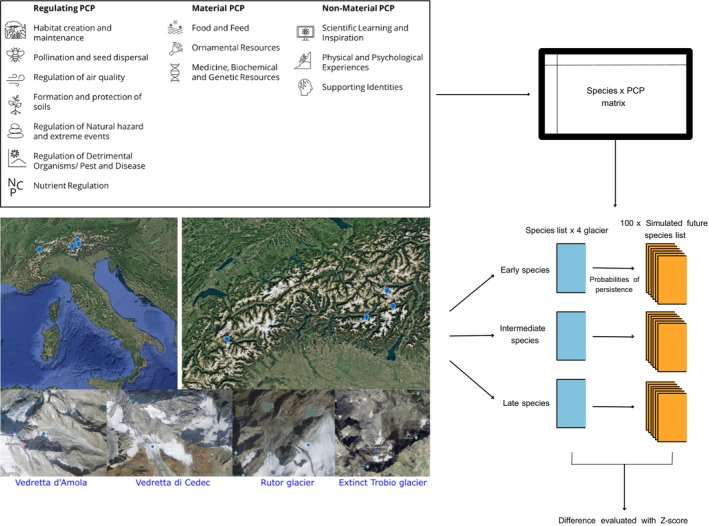
Conceptual framework of the study. The map of the four glaciers in the Italian Alps, the 13 NCPs used for the study, and a summary of the workflow.

This study was conducted in four glacier forelands located in the Italian Alps: Vedretta d'Amola glacier, Western Trobio glacier, Rutor glacier, and Vedretta di Cedec glacier (Losapio et al. 2021) (Figure 1). These sites represent distinct stages of glacier extinction and offer a unique opportunity to assess biodiversity and PCP over a range of different environmental conditions. The four study sites were selected based on a combination of glaciological, climatic, biogeographic, and ecological attributes to ensure a diverse and representative yet comparable assessment of PCP responses to glacier extinction. Each site represents a different stage of glacier retreat and extinction: Western Trobio Glacier underwent a complete extinction in 2023 (Chiarle et al. 2024); the Vedretta d'Amola faces widespread rockfall events that favor the progressive increase in glacier surface debris cover (Chiarle et al. 2024); while Vedretta di Cedec and Rutor Glacier have bigger masses and are retreating fast. While all sites are located in temperate Alpine environments with similar precipitation and mean annual temperature regimes, Amola and Trobio glaciers are located in more peripheral areas as compared to Cedec and Rutor glaciers which are in central Alpine valleys. The lithology of all glacier foreland sites is acidic, and the elevational range is ~1900 to 2600 m a.s.l.

In each site, we established a transect from the glacier terminus (or glacier surface in the case of Vedretta d'Amola debris‐covered glacier) to the grasslands adjacent to Little Ice Age (LIA) moraines. Such transects span terrains from recently ice‐free to thousands of years old, such that plant communities range from 0 (surface of debris‐covered glacier) to ca 10,000 years after glacier retreat. Terrain age was estimated as the mean years since glacier retreat between two moraines (Losapio et al. 2021). The successional stages of glacier retreat were defined as follows: (i) pioneer (i.e., early) stage: 0–50 years post‐glacier exposure as recently deglaciated terrain with sparse vegetation cover; (ii) intermediate stage: 50–150 years, with initial soil development and increasing plant colonization; (iii) late stage: older than 150 years on more developed soils with diverse plant communities. This transect represents a gradient of plant community development after glacier retreat. Furthermore, terrains outside the LIA moraines represent a scenario of glacier extinction, being ice‐free for more than 10,000 years. Terrains inside the LIA moraines undergo primary succession, while communities outside the LIA are more in a steady state, being ice‐free for less than 200 years. Thanks to these two distinct geochronological configurations, here we distinguish between glacier retreat and glacier extinction. With glacier retreat we refer to a situation in which communities are still under the influence of glaciers via bioclimatic effects and geomorphic processes. This situation is assessed along the glacier foreland gradient, within the LIA moraines. With glacier extinction we refer to a scenario in which glaciers have no longer legacy effects on the current communities inhabiting previously glaciated land. This scenario is assessed by comparing the glacier foreland with communities outside the LIA moraines.

Along each transect, three to seven plots of 25–100 m^2^ were randomly placed in each stage of glacier retreat, with the same number of plots per site depending on site size for a total of n=170 plots across the four sites (Losapio et al. 2021), ensuring adequate sampling to assess species persistence and PCP in different successional contexts while covering enough microhabitat heterogeneity and accounting for heterogeneous spatial distributions. We surveyed plant communities by recording the presence/absence of plant species in each plot. The total species pool recorded across all sites consisted of 132 plant species (Table S1). Yet, species with low occurrence frequencies (i.e., occurring only in one plot per site) were excluded from the analyses to ensure robust model predictions. Of the 117 plant species filtered, 99 were finally considered in the following analyses as some species lacked a clear successional classification due to inconsistent occurrence across multiple stages or due to lacking literature data.

### 2.2 Species Persistence Projections Following Glacier Extinction

First, we analyzed plant species' response to glacier retreat by means of a species distribution network approach (Burns and Zotz 2010; Losapio et al. 2019; Marini et al. 2019). We extracted this information from previous results of Losapio et al. (2021). The plant community survey data were transformed into a bipartite network of species distribution over the landscape. A network was built for each site separately. In each network, plant species and community age of each plot are linked by species occurrence, which represents the two parts of the network and the links, respectively. Then, a fast greedy algorithm that optimizes a modularity score (Clauset et al. 2004) was used to analyze community structure identifying modules of plant species that are distributed across stages. Network modules are dense subnetworks characterized by high occurrence frequency of a group of plant species within the same stage and low frequency or no occurrence between other species groups at different stages. This way, each plant species was assigned to one module that corresponds to a glacier retreat stage. Stages can include one or more community ages depending on modularity. Samples species have been finally categorized into early, medium and late.

Second, to predict plant species persistence in a post‐glacier scenario, we employed hierarchical joint species distribution models (HMSC) (Ovaskainen et al. 2017; Tikhonov et al. 2020). This Bayesian framework integrates species occurrence data, environmental factors, and species‐to‐species associations to estimate the probability of species persistence after glacier extinction (Losapio et al. 2021). We modeled species distributions using presence‐absence data from 99 plant species across the four glacier forelands. Estimates of the probability of species persistence after glacier extinction $p_{i}$ were obtained by modeling species distribution in response to environmental conditions (i.e., age, soil texture, soil organic matter), functional traits (i.e., specific leaf area and leaf dry matter content); the co‐occurrence of other species was considered as a latent variable. The glacier retreat spatial gradient served as a temporal proxy for predicting species distributions as older stages represent future deglaciated environments. This chronosequence approach allows us to infer how plant communities might shift after complete glacier disappearance (Ficetola et al. 2021). We considered and extracted the mean posterior probability $\bar{p_{i}}$ for each plant species *i* in the scenario of glacier extinction (Losapio et al.^2021^). We chose not to include climatic variables in our models as climatic variation at the local scale is minimal compared to the dominant effects of glacier retreat and local site conditions such as soil nutrients or neighborhood identity. Glacier retreat was shown to drive non‐linear biodiversity changes, where soil carbon accumulation, physical disturbance, and biotic interactions had greater explanatory power than large‐scale climatic gradients (Caccianiga et al. 2006; Ficetola et al. 2021).

Then, to compare observed PCP with future PCP, we simulated plant communities after glacier extinction on the basis of persistence probability for each plant species. We simulated future plant communities using a binomial distribution $B_{i}\left(n,p\right)$ with $p=\bar{p_{i}}$ for each plant species i independently. We run this simulation for n=100 times generating independent and identically distributed random plant communities composed of *S*_e_ plant species. We can further compare those simulated future plant communities with the current observed plant species *S*_o_.

### 2.3 Plant Species–PCP Relationships

The research is based on a Species by NCP matrix, which reports the contributions provided by every species. We built the matrix by putting the species as lines and the NCP as columns. The contribution values chosen are the following: −1 (negative), 0 (neutral) and 1 (positive). The full table is available in Data S2.

The selection criteria for PCP took into account first the standard list of NCP approved by IPBES (Intergovernmental Science‐Policy Platform on Biodiversity and Ecosystem Services, 2019) and already used in other publications (de Groot et al. 2002; Brauman et al. 2020; Díaz et al. 2018). However, to define PCP that could fit with and provide meaningful ecological information for Alpine plants, we had to partially edit or remove some PCP. As we considered single species, we had to exclude a priori the PCP related to habitats and not to species. We also removed contributions not related to plants in terrestrial ecosystems like ocean acidification. Ultimately, we retained 13 different types of contributions that belong to three different groups (Table S1):

1. Regulating: Functional and structural aspects of organisms and ecosystems able to change the environmental conditions sensed by human communities and/or provide material or non‐material benefits (Report IPBES—2017). They include the following: habitat creation and maintenance, pollination and dispersal, regulation of air quality, formation and protection of soils, regulation of natural hazards, regulation of detrimental organisms, and nutrient regulation.
2. Material: Substances, objects, or other natural materials and products capable of nourishing the physical structures and infrastructure of human communities (e.g., supply of building materials, food, energy, and ornamental elements) (Report IPBES—2017). They include the following: food and feed, ornamental resources, medicine, biochemical resources and genetic resources.
3. Non‐Material: They contribute on a psychological level (both personal and collective) affecting the quality of life. In many cases, they are subjective elements that may vary according to the culture and traditions of the people associated with them (Report IPBES—2017). They include the following: scientific learning, physical and psychological experiences, supporting identities.

We assessed all PCP values through a deep bibliography research to collect all the information required to attribute the correct value of *Contribution* to all the species for every single PCP. For many PCP we had to extend our research to non‐scientific sources (books, blogs, popular articles etc.). The ecological information required by some PCP has been retrieved from the plant databases “Acta Plantarum” (Italian) and “InfoFlora” (Swiss). More information about the websites and the papers consulted could be found in the Supporting Information: Bibliography S3.

### 2.4 Statistical Analyses

We quantified both the absolute contribution ($C_{\mathrm{Abs}}$) and the relative contribution ($C_{\mathrm{Rel}}$) provided by the plant species for each PCP type. We calculated the current absolute contributions provided by plant species $C_{\mathrm{Abs}}$ by summing the values assigned for each PCP. The relative contribution was obtained by dividing $C_{\mathrm{Abs}}$ by plant species richness. We calculated the future absolute and relative contribution using the same methodology. We applied the calculation on each of the 100 future simulations.

We then calculated both $C_{\mathrm{Abs}}$ and $C_{\mathrm{Rel}}$ for each glacier and each stages for both present and future plant communities. We further combined the four glaciers by averaging their PCP scores for the present contribution and combining the scenarios for future simulations.

Changes in $C_{\mathrm{Abs}}$ therefore depend both on changes in species‐specific contribution as well as on changes in current (*S*_o_) and future (*S*_e_) species richness, respectively. Comparing these indices shows the direct effects of changes in plant composition, NCP types, and species richness. On the contrary, $C_{\mathrm{Rel}}$ is relative to species richness and shows whether the resulting community can sustain the different NCP.

We compared the present and future simulations of absolute and relative contribution using the *Z*‐score principle. We calculated the deviance of the 100 simulations to the present scores as $Z=\frac{p-\bar{f}}{\mathrm{sd}\left(f\right)}$, where *p* is the present value of $C_{\mathrm{Abs}}$, *f* the average value of future simulations $C_{\mathrm{Abs}}$ and sd(*f*) the standard deviation across simulations. We followed the same procedure for $C_{\mathrm{Rel}}$. To assess the significance of the comparison between present and future we calculates *p*‐values as $p=1-\sum_{s}^{i}I\left[H_{\mathrm{fut}}>H_{p}\right]/\left(s+1\right)$, where *s* is the number of simulations, *I*[*H*_fut_ > *H*_p_] is an indicator value that equals 1 if the simulated value is greater than the present value and 0 otherwise across 100 simulations +1 empirical value. Data analyses were done in R versions 4.3.2.

## 3 Results

**FIGURE 2:**
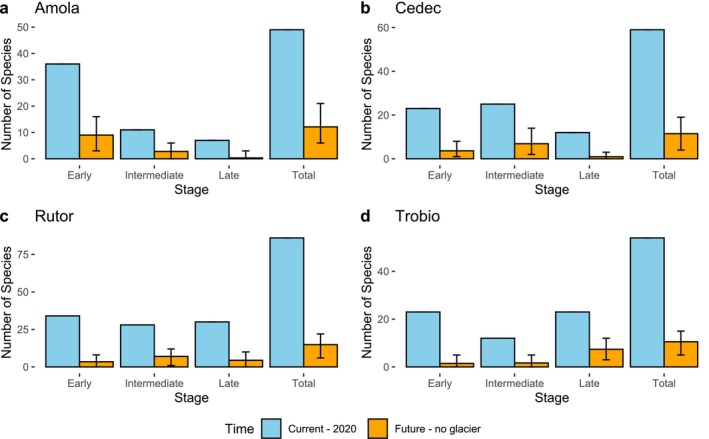
Number of plant species in each glacier and each stage for the present and the future. The blue bar represent the number of species found in the four different glacier forelands. The orange bar represent the average estimated species for the future based on the persistence probabilities and 100 simulations. The error bar represent the lowest and the highest amount found in the 100 simulations.

We observed that plant species richness decreases in future scenarios following glacier extinction (Figure 2). This pattern was consistent across the four glacier ecosystems. Such decrease in plant species richness mirrors the observed decrease in absolute volumes of PCP. On the contrary, changes in relative volumes of PCP does not directly reflect changes in species richness values. For both absolute contributions of plants ($C_{\mathrm{Abs}}$) and relative contributions of plants ($C_{\mathrm{Rel}}$) we present below the results averaged across the four glacier ecosystems sites, including both current assessment and future projections.

### 3.1 Absolute Changes in PCP After Glacier Extinction

**FIGURE 3:**
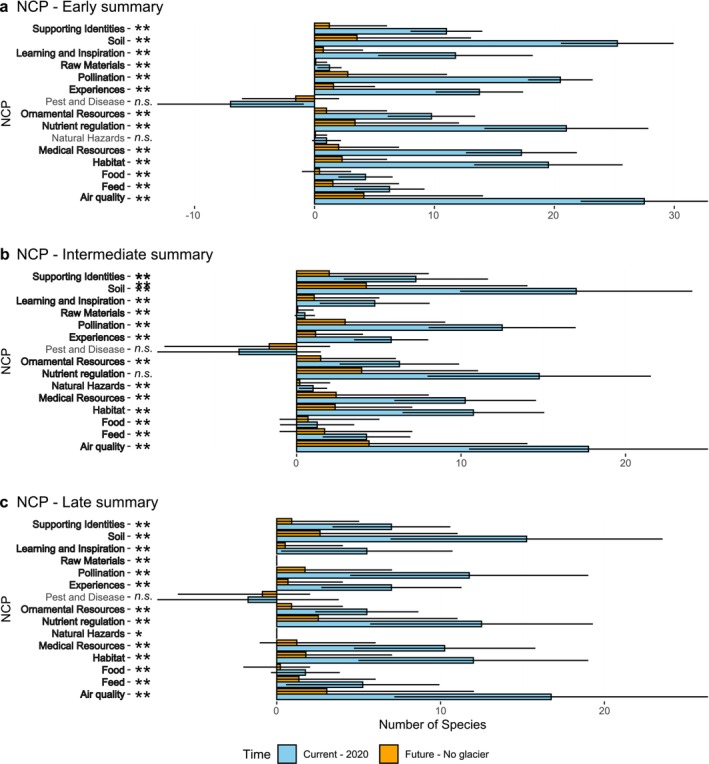
The absolute plant contribution for each NCP. We present the average of the four glaciers for each stage and each NCP. The blue bars represent the current absolute contribution of plants. The error bar represents the standard deviation. The orange bars represent the average simulation for the future. Here the error bar represents the lowest and the highest contribution of the simulation of the four glaciers. The *p* value significance is added on the plot: ns = non‐significant; *p < 0.05; **p < 0.01. In bold, are the NCP that significantly change with glacier extinction.

We found an overall decrease in absolute volumes of PCP following glacier extinction for every species type (Figure 3a). The *p*‐values and *Z*‐scores are reported in Table 1.

**TABLE 1:** *Z*‐score and *p* value of comparison between present and future absolute contribution of plants.

	Habitat c. and m.	Pollination	Air quality	Soil	Natural hazards	Pest	Nutrient R.	Food	Feed	Raw M.	Medial R.	Ornamental R.	Learning and inspiration	Experiences	Supporting identities
Early stage—absolute contribution															
*Z*‐score	**−17.757**	**−19.193**	**−26.056**	**−23.898**	−0.721	6.005	**−19.708**	**−3.679**	**−5.195**	**−0.922**	**−15.950**	**−9.080**	**−10.751**	**−12.343**	**−10.188**
*p*	**0.002**	**0.002**	**0.002**	**0.002**	0.075	1.000	**0.002**	**0.002**	**0.007**	**0.002**	**0.002**	**0.002**	**0.002**	**0.002**	**0.002**
Intermediate stage—absolute contribution															
*Z*‐score	**−9.199**	**−10.955**	**−16.178**	**−15.475**	**−0.557**	2.500	**−13.236**	**−0.584**	**−3.119**	**−0.248**	**−8.889**	**−5.358**	**−3.798**	**−4.373**	**−6.217**
*p*	**0.002**	**0.002**	**0.002**	**0.002**	**0.020**	0.875	**0.002**	**0.002**	**0.002**	**0.002**	**0.002**	**0.002**	**0.002**	**0.002**	**0.002**
Late stage—absolute contribution															
*Z*‐score	**−10.897**	**−10.790**	**−15.699**	**−14.257**	**0.000**	**1.134**	**−11.563**	**−1.332**	**−4.402**	**0.000**	**−9.374**	**−4.675**	**−4.824**	**−6.221**	**−6.182**
*p*	**0.002**	**0.002**	**0.002**	**0.002**	**0.020**	**0.601**	**0.002**	**0.002**	**0.002**	**0.002**	**0.002**	**0.002**	**0.002**	**0.002**	**0.002**

*Note:* In bold, are the NCP that significantly change with glacier extinction.

For pioneer species, we found a significant decrease for most of the contribution types. We observed the largest decrease in contributions to *Air Quality* from 27.50 to 4.11 in average, that is an absolute decrease of Δ=−23.39 (Δ%=−85%) contributing plant species on average across sites. The second most affected PCP is *Soil development* contributions which decrease from 25.25 to 3.54 (Δ=−21.71; Δ%=−86%) plant species on average across sites. The third most affected one is *Nutrient Regulation* contribution from 21.00 to 3.38 (Δ=−17.62; Δ%=−84%) plant species on average across sites. The least affected contributions in absolute terms are *Raw Materials* provisioning, which decreased from 1.23 to 0.10 (Δ=−1.13; Δ%=−92%) while PCP to *Natural Hazards* mitigation showed no significant variation. Notably, the sole increasing PCP was *Pest and Disease* control which increased from −7.00 to −1.56 (Δ=+5.44; Δ%=+79%) plant species on average across sites meaning that less pioneer plant species were found to be negatively associated to pest and disease (Figure 3a).

For intermediate‐stage species, we also found that most of the PCP significantly decreased in absolute volumes (Figure 3b). Similarly to the early stage, we observed the largest decrease of plant species contribution for *Air Quality*, from 17.75 to 4.40 (Δ=−13.35; Δ%=−75%) plant species contributing on average across sites, followed by *Soil development* which decreased from 17.00 to 4.23 (Δ=−12.77; Δ%=−75%) and *Nutrient Regulation* from 14.75 to 3.95 (Δ=−10.80; Δ%=−73%) plant species. The lowest positive PCP and decrease was *Raw Materials* which decreased from 0.50 to 0.06 (Δ=−0.44; Δ%=−88%) plant species on average across sites. We found exceptions for PCP associated with *Natural Hazards* mitigation, which showed no significant variation, and *Pest and Disease* control, which showed a marginal increase from −3.50 to −1.66 (Δ=−1.84; Δ%=+53%).

Similarly, late succession species showed decreasing trends comparable to other species (Figure 3c). We also observed the largest decrease of PCP for *Air Quality*, from 16.75 to 3.05 plant species contributing on average across sites (Δ=−13.70; Δ%=−82%). This one was followed by *Soil development*, with a decrease from 15.25 to 2.64 (Δ=−12.61; Δ%=−83%), and *Nutrient Regulation* from 12.50 to 2.53 (Δ=−9.97; Δ%=−79%). The lowest positive contribution and decrease was *Food provisiong*, which decreased from 1.75 to 0.21 (Δ=−1.54; Δ%=−88%) plant species on average across sites. PCP associated with *Natural Hazards*, *Raw Materials*, and *Pest and Disease* control showed no significant variation.

### 3.2 Relative Changes in PCP After Glacier Extinction

**FIGURE 4:**
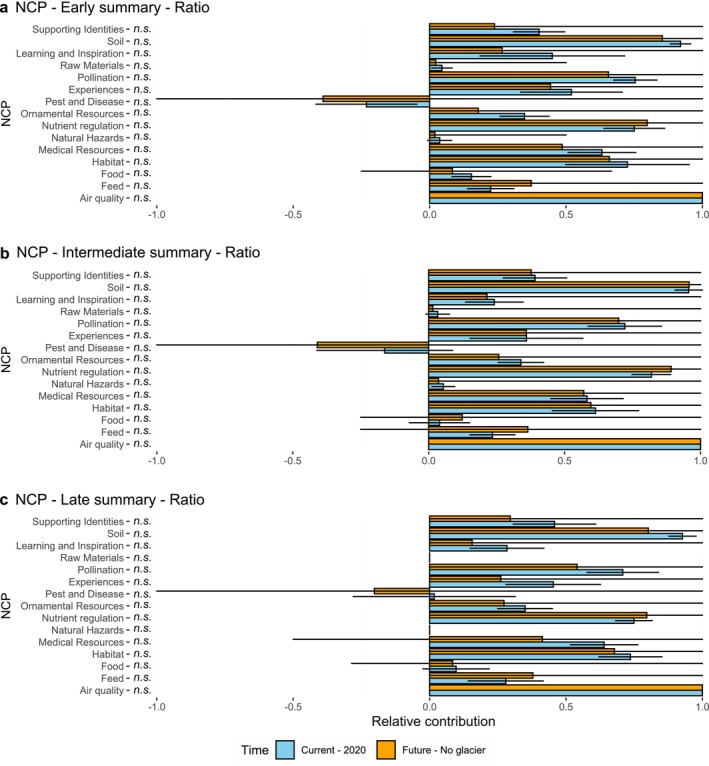
The relative plant contribution for each NCP. Each absolute contribution has been divided by the amount of species present to obtain a ratio between 0 and 1 that represent the relative contribution of plant species to each NCP. We present the average of the four glaciers for each stage and each NCP. The blue bars represent the current absolute contribution of plants. The error bar represent the standard deviation. The orange bars represent the average simulation for the future. Here the error bar represents the lowest and the highest contribution of the simulation of the four glaciers. The *p* value significance is added on the plot: ns = non‐significant.

We further assessed the relative volumes of PCP as a proportion over the total number of plant species recorded for each PCP and stage type, then compared relative PCP changes between the present and the future scenarios following glacier extinction (Figure 4; Table 2). In all three species types, *Air quality* showed the highest representation given by 1 point (i.e., 100%) as all plant species positively contribute to *Air quality*. Then, the most frequent PCP were *Soil and Nutrient Regulation* in the three stages (pioneer, intermediate, and late) for both present and future scenarios (91%; 95%; 92% and 75%; 81%; 74%, respectively). We observed the lowest positive relative PCP for *Natural Hazards* and *Raw Materials* (3%; 5%; 0% and 4%; 3%; 0% respectively). The sole PCP with negative values was *Pest and Diseases*, which shows −23%, −16%, −1.6% for pioneer, intermediate, and late plant species, respectively. Finally, we observed that all PCP showed comparable values between present and future scenarios when considering changes in relative volumes of PCP expressed as a difference in % of plant species.

**TABLE 2:** *Z*‐score and *p* value of comparison between present and future relative contribution of plants.

	Habitat c. and m.	Pollination	Air quality	Soil	Natural hazards	Pest	Nutrient R.	Food	Feed	Raw M.	Medial R.	Ornamental R.	Learning and inspiration	Experiences	Supporting identities
Early stage—relative contribution															
*Z*‐score	1588	1783	Inf	3747	0.237	−0.691	2421	0.425	1069	0.265	1.174	0.401	0.378	0.960	0.591
*p*	0.466	0.344	1	0.504	0.125	0.339	0.653	0.294	0.716	0.150	0.314	0.272	0.287	0.334	0.289
Intermediate stage—relative contribution															
*Z*‐score	1.587	2.171	Inf	7.769	0.294	−0.784	4.088	0.608	1.084	0.148	1.455	0.645	0.732	0.759	0.888
*p*	0.429	0.464	1.000	0.833	0.209	0.289	0.758	0.459	0.711	0.095	0.471	0.399	0.489	0.384	0.536
Late stage—relative contribution															
*Z*‐score	1.784	1.050	Inf	1.704	0.000	−0.420	2.031	0.316	0.963	0.000	0.614	0.688	0.452	0.414	0.603
*p*	0.574	0.461	1.000	0.633	1.000	0.372	0.781	0.456	0.728	1.000	0.426	0.486	0.419	0.424	0.441

## 4 Discussion

Previous studies focused on the services and contributions provided by the glacier on its own or at the coarse landscape scale, neglecting contributions provided by plant diversity associated with glacier habitats. To the best of our knowledge, this study is the first of its kind providing quantitative insights into the impact of glacier retreat on nature's and plants' contributions to people (PCP) on a species‐specific, biodiversity basis. Our findings highlight that PCP decrease in absolute terms with glacier extinction, while relative values of PCP remain similar between present and future communities. This means that changes in PCP are mainly driven by changes in plant species richness. Although future communities might be able to provide contributions in similar proportions as current ones, the total volumes of PCP delivered would be substantially reduced by glacier extinction and inherent biodiversity change.

### 4.1 PCP as a Tool to Assess Challenges and Opportunities After Glacier Extinction

Glacier forelands host some unique specialized plant species (Cauvy‐Fraunié and Dangles 2019; Losapio et al. 2021). Glacial biodiversity is not only important per se or for ecosystem functioning, but is also profoundly embedded in cultures and economies (Petelka et al. 2022; Vitalini et al. 2013). Plants sustain life and life support systems on planet Earth and constitute a fundamental living resource for human life and economic activities (DelSesto 2020; Roches et al. 2021; Bar‐On et al. 2018). As we showed here, plants in Alpine environments—but not only—provide a broad range of contributions to people supporting human well‐being. These PCP range from better air quality and soil development to relevance in medicine and food consumption (Diazgranados et al. 2020; Howes et al. 2020; Molina‐Venegas et al. 2021; Sia et al. 2023).

In accordance with other studies, our results firstly pointed out the consequences of a decrease in plant species richness following the extinction of glaciers (Cauvy‐Fraunié and Dangles 2019; Losapio et al. 2021). The trend is found for the four studied glaciers and the three different stages. With the decline of plant species in the future we are also facing the risk of a reduction of contributions to people provided by plants. On one hand, we found that future communities are able to sustain PCP levels at the same proportions as today as glacier extinction may not affect the relative PCP. On the other hand, we highlighted that the amount of the majority of PCP drastically decreases with the decrease of plant richness as glaciers go extinct. Combining both results suggests that PCP are sustained by the remaining plant species in future communities, but they are supported by lower richness levels. This suggests a possible risk of having more homogenized and poorer PCP. As an example, PCP such as *Medical Resources* or *Raw materials* are supported by just one last remaining plant species as opposed to as much diversity as five or more species. These PCP supported by solely one remaining species are therefore at the edge of getting locally lost. We highlight that the impoverishment of communities makes PCP more vulnerable and unstable (Molina‐Venegas et al. 2021).

In addition to glacier extinction, Alpine plants will face a range of different perturbations and challenges in the near future, such as the migration to higher altitudes of shrubs and trees, reduction of snow cover, or simply temperature increase (Chauvier‐Mendes et al. 2024; Rumpf et al. 2018; Alexander et al. 2015). At the examined study sites, such additional perturbations could contribute to the local extinction of some of the last remaining plant species, leaving an uncertain future concerning some of the contributions to people provided by plants. For example, *Artemisia genepi*, a species supporting various PCP, is present in only a few of the 100 simulations. In addition, some PCP, such as *Learning and inspiration* are supported by only a single species—varying depending on simulations—or none at all, indicating that this NCP is highly threatened and at risk of disappearing from glacier forelands.

Our case study highlights, among others, a potential loss of plants used for food resources for both animals (*Feed*) and humans (*Food*). We observed a 50% decline of PCP related to both feed and food. Today, most of the population is fed with only a few crop types (Massawe et al. 2016; Renard and Tilman 2021). However, wild plants can still represent an important source of food production for both local populations and for increasing diet diversity (Massawe et al. 2016). Local wild plants play a crucial role in ensuring pasture stability (Sonnier et al. 2024). In the coming years, as glaciers continue to retreat, mountain regions will face, in addition, increasingly extreme climatic events, posing a significant threat to plant communities and, consequently, to cattle feed (Sonnier et al. 2024). Maintaining greater plant diversity enhances pasture resilience, allowing ecosystems to better withstand environmental stressors (Sonnier et al. 2024). Moreover, diverse plant communities provide greater temporal variability in forage availability (Zimmer et al. 2022), ensuring cattle can be fed freely over longer periods. In addition, the local flora plays a vital role in regional dairy and meat production (Martin et al. 2005). Many Alpine regions are renowned for their unique cheese varieties, which range from locally appreciated specialties to internationally recognized products (Martin et al. 2005). These cheeses not only contribute significantly to the local economy, but also attract tourism, strengthening the cultural and economic ties of the region to its traditional agricultural practices (Grandini et al. 2022). However, with the loss of plant diversity shown in our results, some of those practices and local heritage could be at risk as many Alpine meadows and vegetations are under the influence of glacial ecosystems. Protecting glacier forelands, in addition to being necessary morally and ecologically, also represents an opportunity for food security. Promoting the extensive cultivation of a variety of local wild plants could simultaneously enhance biodiversity, support local farming, and boost regional economies while improving local food security.

One way to enhance biodiversity in glacier forelands could be through the implementation of sustainable farming practices. For example, in the Andes, the introduction of Lamas (*Llama glama*) has increased plant diversity in a glacier foreland and enhanced diversity during primary colonization (Zimmer et al. 2023). Low cattle density is known to promote soil development by both trampling and increasing soil nutrients through their waste and to favor dispersion through the transport of seeds and pollen (Zimmer et al. 2023). In addition, cattle can reduce shrub encroachment that happens in late colonization (Erschbamer and Caccianiga 2016) and promote species diversity associated with pasture (Losapio et al. 2024). However, intensive pasture could disrupt the vegetation by modifying soil properties, with important input of nutrients (Jewell et al. 2007).

The loss of certain PCPs could trigger cascading effects on ecosystem functions. For example, the significant decline of herbaceous plant species that support pollinator communities in our study could lead to a reduction in pollination services (Inouye 2020; Ollerton 2017; Vasiliev and Greenwood 2020). Previous studies in glacial ecosystems have documented a decrease in pollinator diversity following plant diversity loss due to glacier retreat (Conti et al. 2025), underscoring the importance of floral diversity in sustaining pollinator communities (Tu et al. 2024). A decline in pollinator populations can severely disrupt ecological networks and trigger secondary extinctions (Harvey et al. 2023; Conti et al. 2025). To counterbalance such biodiversity loss and prevent ecosystem function erosion, significant human and financial resources would need to be allocated (Murphy et al. 2022; Ollerton 2017; Vasiliev and Greenwood 2020). These examples highlight the importance of glaciers to sustain PCP, the potential consequences of PCP loss, and the importance of enhancing plant biodiversity.

### 4.2 Limitations and Perspectives

We provide a scalable and adaptable framework for assessing the species‐specific contributions provided by diverse species and plant diversity across communities. This case study also warns of a potential erosion in ecosystem services and NCP following glacier extinction, providing valuable information about the PCP most critical in the near future. Within this framework, one can now track back the plants most central to supporting PCP across categories and communities. However, some important components are still missing to complete the picture and implementation.

First, as we considered “only” the probability of plant species persistence at each stage, we did not include potential new plant species invading glacier forelands. Although we did consider the “climax” vegetation as the reference stages toward which communities tend to develop after glacier extinction (Erschbamer and Caccianiga 2016; Ficetola et al. 2021) and from which plant species currently occurring at late‐successional stages could colonize and invade pioneer and intermediates stages in the future, we did not consider plants originating from lower altitudes such as from treeline forests and mowed meadows. Using broad‐scale species distribution models may help to map future distributions of plant species while including new potential “invasive” species (Adde et al. 2023). Such modeling could further accurately map PCP in glacier forelands for the present and the future. Future studies shall also include upward colonization of lowland species or invasive species currently not occurring in glacial landscapes.

Second, this study is based on presence–absence data. Consequently, information on species abundance and biomass has been overlooked. This also implies that all plant species have the same species‐specific PCP weight. We are confident that presence–absence data provide reliable estimates for some regulating and non‐material PCP, such as *Pollination* or *Supporting identities*, for which species abundance or biomass could be neglected to some extent. Instead, quantitative data on abundance, biomass, or cover may improve the estimation of material PCP such as *Air quality* or *Formation and protection of soil*. As every species has a positive impact on air quality, a higher plant cover might be more beneficial to a better air quality in addition to diversity. Future studies shall direct effort toward developing methods for combining predictive models (e.g., HMSC, SDM) with abundance data and weighted PCP.

Protecting glacier forelands requires considerable time and effort, including land allocation, regulation, and equitable resource management to balance conservation goals with existing land uses. We based our study on an extensive literature review and research, but it is known that incorporating local knowledge is essential to fine‐tuning with real‐world cases to better understand how these areas are currently used and how they might be utilized in the future. Ultimately, integrating local knowledge and focusing on the most vital and threatened PCP can support effective conservation strategies in glacier forelands by ensuring that these endangered environments are managed sustainably and equitably for the benefit of both ecosystems and local communities (Zimmer et al. 2022). Such insights would improve conservation efforts, ensuring they are both effective and fair (Huggel et al. 2019; Tucker et al. 2021; Zimmer et al. 2022).

Our case study focuses on four Italian glacier sites, offering a localized perspective that is crucial for effective decision‐making. These glaciers and their forelands are critically endangered environments, as exemplified by the fact that one glacier (Trobio glacier) is already extinct as of 2023 (Chiarle et al. 2024) while a second one (the Vedretta d'Amola) may undergo extinction in the next decade. Yet, as new ecosystems emerge from glacier retreat, these areas are increasingly utilized by humans (Zimmer et al. 2022) for a diverse range of activities, including tourism, recreation, sport, farming, energy production, and notably scientific research. This picture highlights the dualism between threats of glacier extinction and socio‐economic possibilities, further mirroring a growing gap between fundamental research and the practical implementation of conservation measures. Local and small‐scale studies, such as this one, are valuable in bridging this gap by providing more precise quantification of NCP that practitioners can further use to guide targeted, ad‐hoc management strategies (Olander et al. 2017). Evidently, this case study could be replicated and extended to other sites, while our PCP framework can be implemented and scaled for environments where biodiversity‐based ecosystem services need to be assessed, such as Polar Regions or glacier‐fed rivers and lakes. Such future global studies and assessments of PCP would be informative to understand the global trends in ecosystem services in the face of glacier retreat and glacier extinction.

In conclusion, by developing a novel framework based on plant diversity, we could illustrate that the consequences of climate change for ecosystem services and NCP are far‐reaching, influencing diverse and broad environmental functions with socio‐economic implications. Sharing the consequences of biodiversity loss can potentially make people more concerned about the current biodiversity crisis of the Anthropocene. The variety of plant species providing each PCP highlights the need to enhance plant biodiversity as well as to sustain all positive contributions provided by plants for people and the planet.

## Conflicts of Interest

The authors declare no conflicts of interest.

## Supporting information


Data S1.

.

## Data Availability

Data and R script are publicly and freely available on Zenodo at https://doi.org/10.5281/zenodo.15222353.
