# Supplementary material for: Plants' Contributions to People Shift With Glacier Extinction
Source: Plant Environ Interact. 2025 Apr 18;6(2):e70041. doi: 10.1002/pei3.70041 (PMC12006824; doi:10.1002/pei3.70041)
Supplement: Supplementary file 1 — Data S1. [file PEI3-6-e70041-s002.docx]

## *Plant-Environment Interactions* Supporting Information

Article title: Plants’ contributions to people shift with glacier retreat

Authors: Nora Khelidj, Simone Balestra, Marco Stefano Caccianiga, Bruno E.L. Cerabolini, Duccio Tampucci, Gianalberto Losapio.

The following Supporting Information is available for this article:

**Dataset S1** Plant-PCP relationship table. Species x PCP matrix. With the stage information in addition. We showed the 16 PCP, however we decided to exclude three of them : *Climate regulation, Water supply,* and *Energy* because they were linked to landscape feature and not to the species properties.

Table explanation: 1 indicate a positive relationship between the plant species and the PCP, 0 is a neutral relationship between the plant species, and -1 is a negative relationship between the plant and the PCP.

There is the list of the 132 species that were observed in the four glacier sites. Plants with only one occurrence plot were excluded, we further excluded plants with incomplete PCP (blank space).

The column stage presents three variables: P = Pioneer, I = Intermediate, L = Late. Those are the ecological stage assigned after the modularity calculation.

**Table S2** Final PCP table and their descriptions.

The 16 PCP are presented and separated in 3 different groups: Regulating, Material, Non-Material.

For each NCP we provide a brief explanation, the description applied for alpine plants. The methods we used to assign 1,0 and -1 to each plant, as well as the source for the NCP description.

**Methods S3** Bibliography of the PCP-table relationship.

The bibliography is organised by group of PCP and for each group the bibliography is listed. When NA is indicated it means that the information was not retrieves on scientific papers, but rather from website. This is specified in the Table S2.
